# Supplementary material for: Transmembrane stem factor nanodiscs enhanced revascularization in a hind limb ischemia model in diabetic, hyperlipidemic rabbits
Source: Sci Rep. 2024 Jan 29;14:2352. doi: 10.1038/s41598-024-52888-6 (PMC10825164; doi:10.1038/s41598-024-52888-6)
Supplement: Supplementary file 1 — Supplementary Figures. [file 41598_2024_52888_MOESM1_ESM.docx]

**Supplementary Figures**


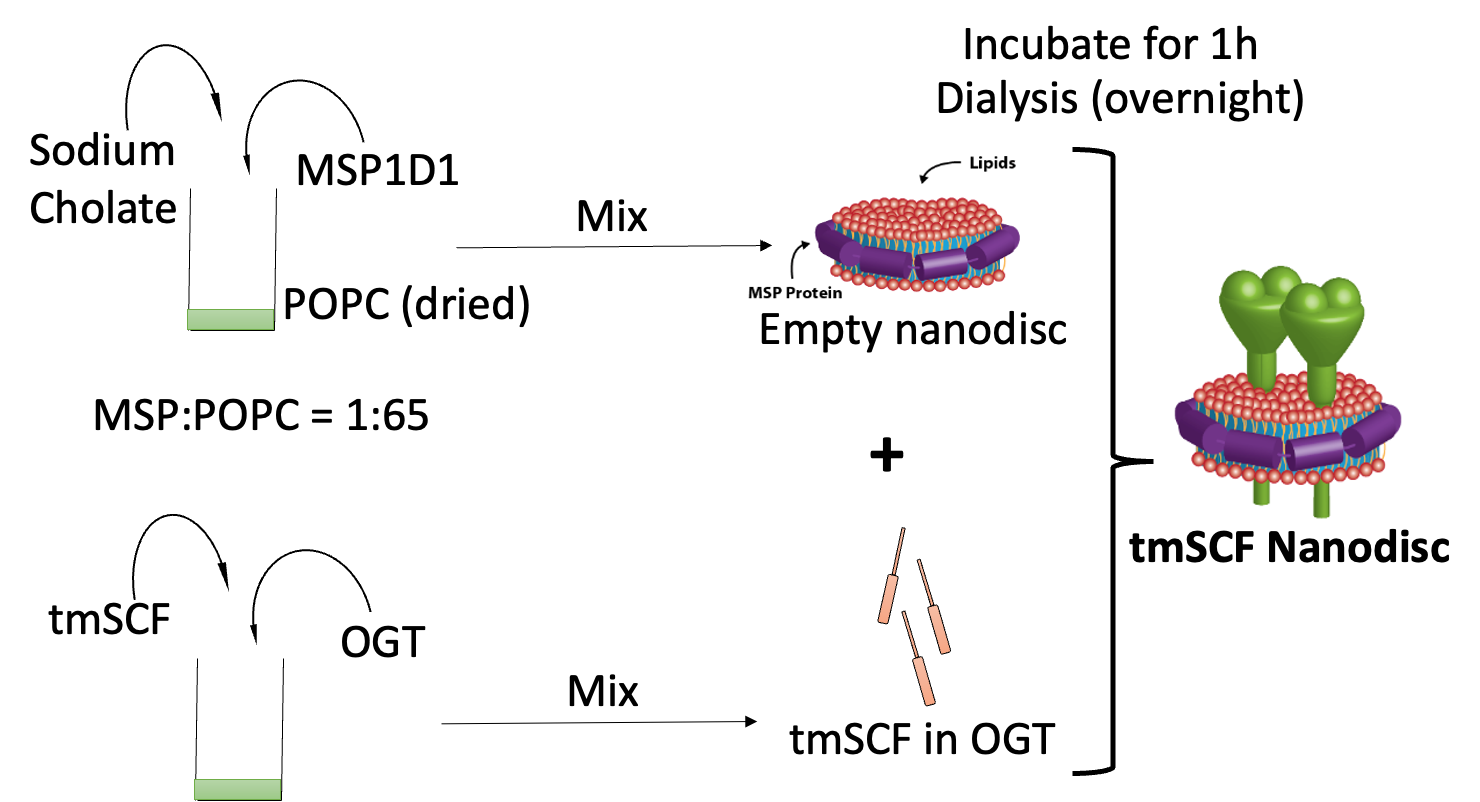


**Supplemental Figure 1.** Schematic illustration for fabricating tmSCF nanodiscs.

**
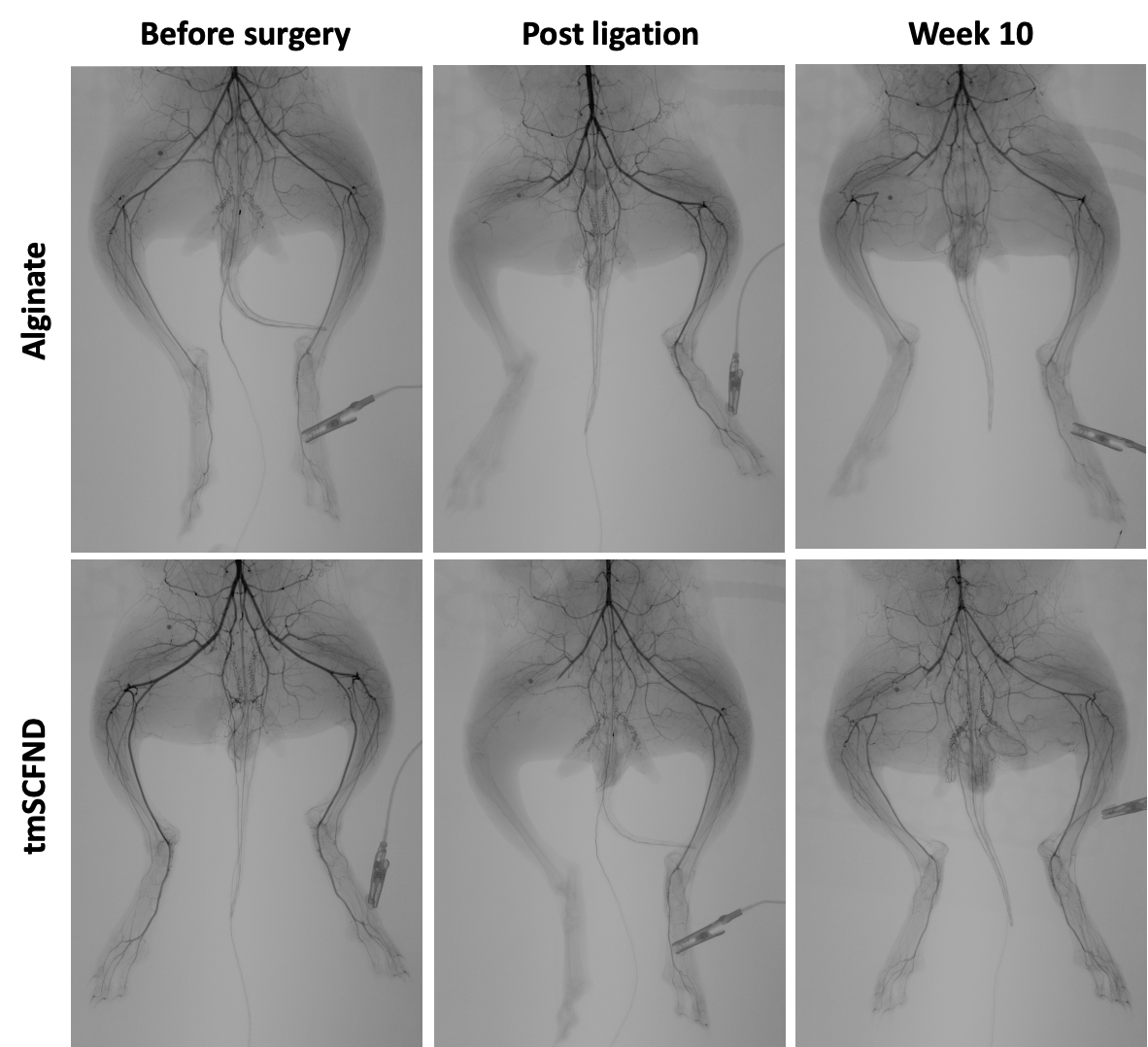
**

**Supplemental Figure 2.** Full angiograms for the rabbits before/after ligation and 10 weeks post-surgery.
